# PMSPA-Net framework with geometric region decomposition for high-precision phenotyping of field-grown maize seedlings

**DOI:** 10.3389/fpls.2026.1920542

**Published:** 2026-09-14

**Authors:** Jing Zhou, Wenlong Zou, Yushan Wu, Helong Yu, Mingren Cui, Jianyu Lu, Lixin Hou, Yijia Tang, Yudi Gao, Min Wu, Yuntao Ma, Jian Zhang

**Affiliations:** 1 College of Information and Technology, Jilin Agricultural University, Changchun, China; 2 Faculty of Agronomy, Jilin Agricultural University, Changchun, China; 3 College of Information and Electrical Engineering, China Agricultural University, Beijing, China; 4 Department of Biology, University of British Columbia, Okanagan, Kelowna, BC, Canada

**Keywords:** field-grown maize seedlings, geometric region decomposition, high-precision phenotypic analysis, PMSPA-Net, point-cloud segmentation

## Abstract

**Introduction:**

Accurate extraction of maize seedling phenotypes is essential for early growth assessment, yet field-grown seedlings exhibit complex backgrounds, non-rigid leaf deformation, self-occlusion, and adhered leaves that challenge three-dimensional point-cloud analysis.

**Methods:**

Here, we developed a workflow integrating PMSPA-Net, an enhanced PointNet++ model, with geometric region decomposition for semantic and organ-level segmentation of field-grown maize seedlings. Multi-view RGB images of 65 complete plants were reconstructed with OpenMVS, and all data were partitioned at the whole-plant level before point-block sampling. Fifteen plants were retained as a fixed independent test set, while the remaining samples were used for training and validation across three predefined random seeds.

**Results:**

Under a common protocol, PMSPA-Net outperformed PointNet, PointNet++, DGCNN, and RandLA-Net, achieving an mIoU of 0.8286 ± 0.0149, a macro-F1 of 0.8981 ± 0.0238, and a balanced accuracy of 0.9561 ± 0.0223. A four-configuration ablation study showed complementary gains from the multi-scale spatial pyramid attention module and IOA-based hyperparameter optimization. Geometric region decomposition also achieved more accurate and complete adhered-leaf separation than DBSCAN in three annotated cases. Point-cloud-derived leaf length and width agreed well with manual measurements.

**Discussion:**

This workflow demonstrates the feasibility of integrated semantic and organ-level phenotyping under the evaluated field conditions; validation across additional sites, developmental stages, and acquisition conditions is required before broader generalization.

## 1 Introduction

As one of the top three staple crops globally, maize plays a critical role in sustaining the world’s food supply (Erenstein et al., 2022). The growth status of maize during the seedling stage directly affects its final yield and quality (Sun et al., 2021). Therefore, selecting phenotypically superior plants during the seedling stage is of significant importance for breeding high-yield, high-quality varieties in the subsequent stages. The overall growth trajectory of maize during this period fundamentally depends on the efficiency of photosynthesis (Urban et al., 2021). As the key organ responsible for this vital process, maize leaves’ morphological traits (such as leaf length, width, area, curling, leaf edge contour, and leaf margin color) not only provide a direct reflection of the plant’s photosynthetic efficiency but are also closely related to its genetic makeup. These traits are essential criteria for assessing yield potential and quality characteristics in maize breeding and phenotypic research (Peng et al., 2021; Wu et al., 2022a). Traditional methods of extracting leaf phenotype parameters typically rely on manual measurements or 2D image-based techniques, which are not only labor-intensive but also inefficient (Akhtar et al., 2024). Furthermore, these methods are susceptible to occlusion effects and may even disturb the plant’s growth dynamics, ultimately impacting its growth potential (Pierik and Ballaré, 2021). Consequently, traditional phenotyping methods struggle to meet the growing demand in modern agriculture for high-precision, detailed phenotypic analysis. To overcome these limitations, in recent years, three-dimensional imaging technologies in high-throughput plant phenotyping, such as laser scanning, multi-angle imaging, and LiDAR, have gained increasing attention from plant phenotyping researchers (Guo et al., 2021; Zhang et al., 2023a, b). 3D data not only provides accurate 3D coordinate information but also rich texture and structural details (e.g., color and normal vectors) (Yan et al., 2024), laying a solid foundation for extracting complex and diverse phenotypic parameters.

However, current research on plant point cloud data has primarily focused on separating multiple plants into individual specimens (Fanigliulo et al., 2020; Mitsanis et al., 2024), while fine-scale segmentation of organs within a single plant and the subsequent extraction of detailed digital phenotypic parameters remain significantly underexplored. For example, in the complex and dynamic conditions of field environments, accurately segmenting plant organs from collected 3D point cloud datasets remains a challenging task (Patel et al., 2023). To address this, researchers have employed various methods based on thresholding, geometric features, and 3D skeletonization to improve the accuracy of plant organ segmentation and phenotypic parameter extraction (Yao et al., 2025). Nevertheless, these approaches often require extensive manual intervention and intricate parameter tuning, which limits processing efficiency. Therefore, there is a pressing need for novel methods capable of achieving efficient and accurate organ segmentation of single-plant 3D point cloud data under complex field conditions, to meet the demands of high-precision and automated analysis. With advances in high-performance hardware and neural network architectures, integrating deep learning techniques with image processing methods for 3D point cloud data has shown tremendous potential in improving the generalizability and accuracy of plant organ segmentation (Bello et al., 2020; Hazer and Yildirim, 2022; Ding et al., 2023). PointNet and PointNet++, as pioneering end-to-end deep learning architectures, operate directly on raw point cloud data to achieve object classification and part segmentation tasks (Zhou and Tuzel, 2018; Jiang et al., 2022). As attention mechanisms continue to evolve, numerous studies have introduced diverse attention modules tailored to the unique characteristics of different datasets, aiming to enhance the overall performance of these networks (Brauwers and Frasincar, 2023).

Additionally, another challenge in the field of plant phenotypic parameter extraction is the lack of high-quality open-source datasets for training and evaluating plant point cloud organ segmentation methods (Luo et al., 2023; Guo et al., 2023). Acquiring 3D point cloud datasets related to plants is particularly difficult, as it not only requires appropriate data acquisition equipment and methods, but the complex structural characteristics of plants also increase the need for extensive manual annotation of the datasets (Scharr et al., 2016; dos Santos Ferreira et al., 2019; Greenwald et al., 2022). High-quality point cloud datasets can significantly enhance the segmentation performance of deep learning models (Li et al., 2022; Zhang et al., 2022). Therefore, constructing high-quality 3D plant point cloud datasets has become a central focus in the field of plant point cloud organ segmentation research (Liu et al., 2025).

To address the limitations of previous studies in field-based 3D acquisition, semantic organ segmentation and adhered-leaf separation, this study proposes an enhanced PointNet++ framework integrated with geometric region decomposition. Specifically, the study pursues three objectives: (1) to construct a field maize seedling point-cloud dataset using multi-view imaging and OpenMVS reconstruction; (2) to evaluate PMSPA-Net, incorporating Multi-Scale Spatial Pyramid Attention (MSPA) and Ivy Optimization Algorithm (IOA)-based hyperparameter optimization; and (3) to separate adhered leaves and extract supported organ-level phenotypic descriptors.

## 2 Materials and methods

**Figure 1** summarizes the study workflow, which comprised three stages: data acquisition and processing, methodological design, and phenotypic trait extraction. First, multi-view images were acquired using a portable 3D scanning system and reconstructed into high-quality colored point clouds. Second, manually measured leaf traits were used as reference data, while PMSPA-Net and geometric region decomposition were employed for semantic and organ-level segmentation. Third, the segmented leaves were flattened and analyzed to extract multidimensional phenotypic traits.

**Figure 1:**
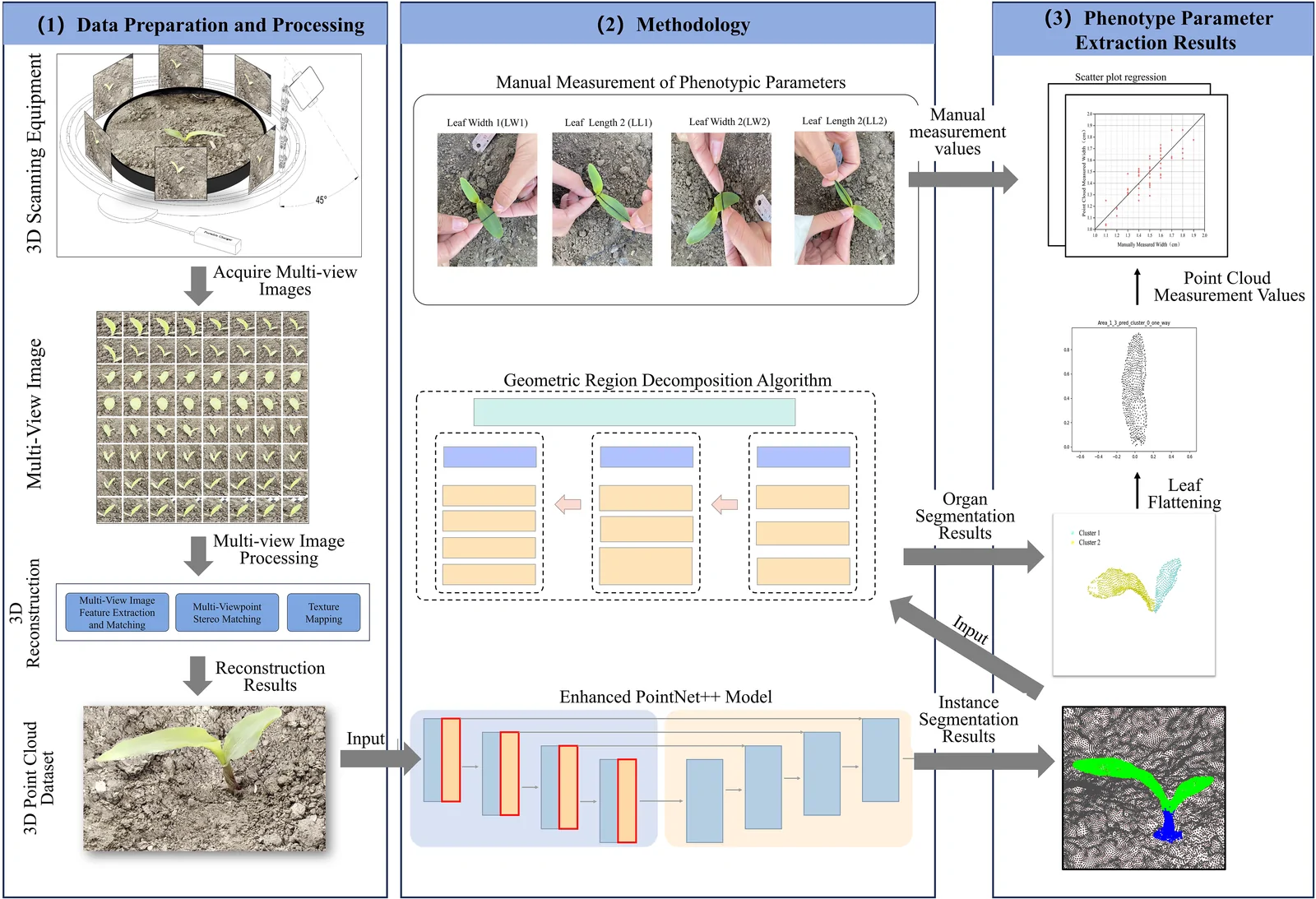
Overall technical workflow of this study.

### 2.1 Multi-view image data capture

Field experiments were conducted at the Jilin Agricultural University Teaching and Research Base in Changchun, Jilin Province, China (43°49′N, 125°24′E), to acquire multi-view images of maize seedlings. The study site is shown in **Figure 2A**, and the field layout is shown in **Figure 2B**. The study area lies in the Northeast Plain and has a temperate continental monsoon climate, with warm, humid summers, cold, dry winters, and short spring and autumn seasons. The mean annual temperature is 4.8 °C, annual precipitation ranges from 522 to 615 mm, and annual sunshine duration is approximately 2,688 h. Images were acquired between 15:00 and 18:00 in June 2024 under clear weather. Plants were arranged with approximate within-row and between-row spacings of 0.40 and 0.50 m, respectively. Although these nominal spacings correspond to a theoretical density of 5 plants m^−2^ under an ideal rectangular layout, the actual field density, calculated from the number of established plants within the effective planted area, was approximately 7 plants m^−2^. Seedling-stage phenotyping was selected because early morphological traits provide useful indicators for breeding selection and field management.

**Figure 2:**
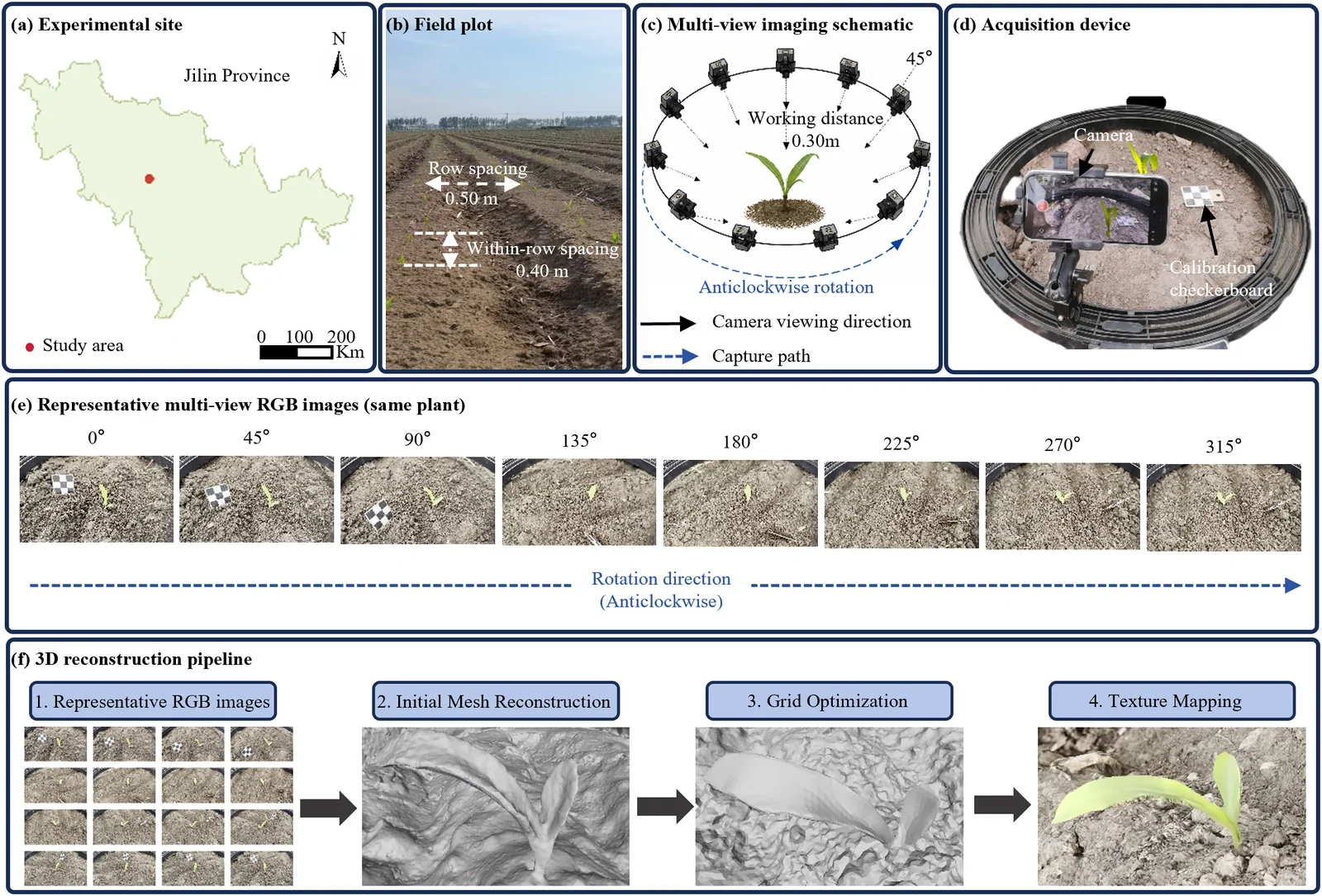
Multi-view image acquisition and OpenMVS reconstruction workflow for field-grown maize seedlings. **(A)** Location of the experimental site in Jilin Province, China. **(B)** Field plot with 0.50 m row spacing and 0.40 m within-row spacing. **(C)** Multi-view imaging geometry showing the 45°camera angle, 0.30 m working distance, and anticlockwise rotation. **(D)** Physical acquisition device with camera and calibration checkerboard. **(E)** Representative image sequence acquired during rotation. **(F)** Reconstruction pipeline from RGB images to initial mesh reconstruction, grid optimization, and texture-mapped 3D plant model.

Unlike fixed-angle crop photography, the acquisition system captured multi-view images to recover plant structure from multiple viewpoints. We designed a portable 3D scanning setup (**Figures 2C**, **D**) comprising an imaging device, portable power supply, electric turntable, device stand, and calibration checkerboard. The camera was positioned at a 45° downward angle with a working distance of 0.30 m, and the turntable provided anticlockwise coverage around each seedling. This configuration reduced interference from neighboring plants and weeds while preserving the visible morphology of leaves and stems. Ten genetically distinct maize genotypes were sampled at the seedling stage, yielding 65 high-quality point-cloud datasets. Each plant was imaged for approximately 1–2 min, producing about 90 views for reconstruction (**Figure 2E**). Leaf length and width were then measured manually with cotton thread as reference data for evaluating the subsequent trait-extraction pipeline.

### 2.2 3D point-cloud construction

Multi-view stereo (MVS) reconstructs detailed 3D models from overlapping images and is widely used in computer vision and digital agriculture (Fuhrmann et al., 2015). OpenMVS is an open-source MVS library that integrates a complete 3D reconstruction workflow. In this workflow, structure from motion (SfM) estimates camera poses and sparse geometry, whereas MVS generates dense surface information from matched image features (Kholil et al., 2021).

OpenMVS was used to reconstruct colored 3D point clouds from the multi-view image sequences. The workflow first used structure from motion (SfM) to extract and match image features, estimate camera poses, and generate a sparse point cloud. The sparse reconstruction and camera parameters were then passed to the multi-view stereo (MVS) module to produce a dense cloud with improved geometric detail. Mesh optimization and texture mapping were applied to preserve leaf morphology and surface color, as illustrated in the reconstruction pipeline in **Figure 2F**. The final PLY point cloud was represented as an M×6 array, where M denotes the number of points and each row stores 3D coordinates (X, Y, Z) and RGB values (R, G, B).

### 2.3 3D point-cloud segmentation

#### 2.3.1 3D point-cloud preprocessing

The maize point-cloud models generated with OpenMVS were highly dense, with each 3D model typically containing approximately 250,000 to 270,000 points (Wu et al., 2022b). These point clouds retained plant structure, field background, and a small amount of noise, indicating that OpenMVS reconstruction provided sufficiently complete data for downstream analysis. Downsampling is commonly used to reduce computational cost in point-cloud processing (Xiang et al., 2022), but excessive downsampling can remove structural details needed for accurate trait extraction (Qiao et al., 2023). Because the reconstructed point clouds already had sufficient completeness and density, the original OpenMVS outputs were used directly for subsequent segmentation and phenotypic trait extraction.

Point-cloud annotation is labor-intensive, and current studies often rely on open-source annotation tools (Ibrahim et al., 2021). We used CloudCompare to assist point-cloud segmentation and annotation because it supports point-cloud processing, segmentation, registration, and measurement (Valero et al., 2020). Each maize point cloud was annotated into three semantic classes: background, leaves, and stems. The dataset followed the general format of the S3DIS benchmark (Armeni et al., 2016), with point coordinates and corresponding class labels stored for model training and evaluation. In total, the dataset contained 65 complete maize point-cloud models and 195 annotation files.

To prevent information leakage between the training and evaluation data, dataset partitioning was performed at the whole-plant point-cloud file level before point-block sampling. Of the 65 complete maize point-cloud samples, 15 were randomly selected once and retained as a fixed independent test set. The remaining 50 samples were divided into training and validation sets at a 4:1 ratio, yielding 40 training samples and 10 validation samples. The training–validation split and model training were repeated using three predefined random seeds to assess the robustness of model performance. Point-block sampling was conducted independently within each subset after the file-level partitioning, ensuring that blocks derived from the same plant did not appear in different subsets.

#### 2.3.2 PMSPA-Net model construction

PointNet is a deep learning model specifically designed for processing 3D point cloud data. By individually processing each point and aggregating global features, it was the first to achieve efficient point cloud segmentation and classification (Qi et al., 2017a). However, PointNet exhibits certain limitations in capturing local geometric information due to its insufficient ability to represent neighborhood features, which may result in the loss of fine-grained details (Liu et al., 2019). To address this issue, Charles R. Qi proposed PointNet++ (Qi et al., 2017b). PointNet++ introduces the Set Abstraction (SA) layer and Feature Propagation (FP) layer, and adopts Multi-Scale Grouping (MSG) and Multi-Resolution Grouping (MRG) strategies to more effectively extract both local and global features of point clouds, thereby enhancing its capability to handle complex 3D point cloud processing tasks. Nonetheless, PointNet++ still faces certain limitations in feature extraction. On one hand, PointNet++ relies on local neighborhood queries and downsampling operations, which may lead to partial loss of point cloud features—particularly when dealing with complex geometric structures or long-range dependencies (Yu et al., 2024a). On the other hand, the inherent sparsity and unordered nature of point cloud data make it challenging to model global contextual information, which constrains the model’s performance in certain complex scenarios (Zeng et al., 2024).

To better capture point cloud features and achieve semantic segmentation of maize point clouds, this study introduces an optimized architecture based on PointNet++, incorporating a Multi-Scale Spatial Pyramid Attention (MSPA) module. The overall network architecture and the integration of MSPA into PointNet++ are illustrated in **Figure 3**. The MSPA module is designed to enhance the model’s ability to extract multi-scale features and improve its performance in modeling global contextual information. MSPA is a lightweight and high-performance attention mechanism that leverages both local and global features from the input point cloud. It extracts enhanced features through a sequence of convolutional layers, normalization layers, and activation functions, thereby strengthening the model’s capacity to capture detailed point cloud features (Yu et al., 2024b). The MSPA module consists of three main components: the Hierarchical-Phantom Convolution (HPC) module, the Spatial Pyramid Recalibration (SPR) module, and a Softmax operation. First, the input features are fed into the HPC module, which splits the input along the channel dimension into s sub-feature maps. These are progressively enhanced through hierarchical residual connections to strengthen multi-scale feature representation. All sub-feature maps are then concatenated to form a multi-scale enhanced feature map. Next, the enhanced feature map is passed into the SPR module, where Global Average Pooling (GAP) and Local Average Pooling (LAP) are applied to generate channel descriptors. These descriptors are fused via weighted summation and passed through two point-wise convolutions followed by a Sigmoid activation to generate the final channel attention weights. Lastly, a Softmax operation is applied to normalize the attention weights. The normalized weights are then applied to the corresponding channels via element-wise multiplication, producing the final output feature map.

**Figure 3:**
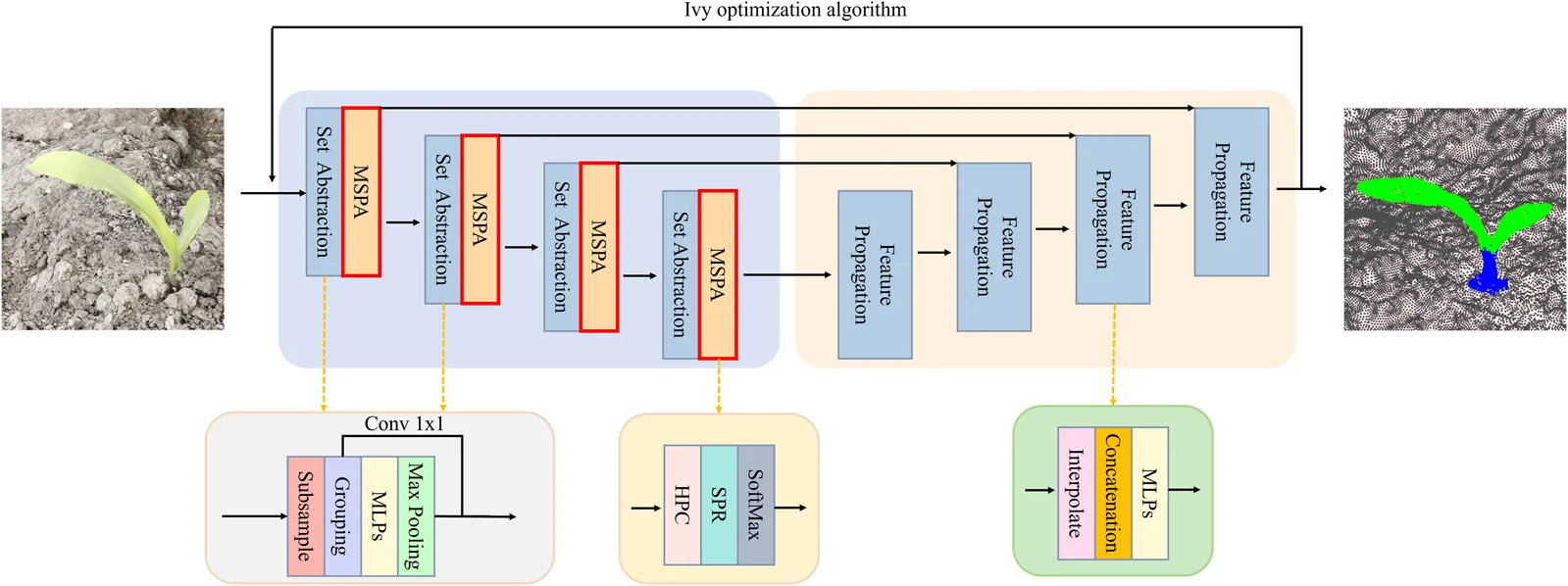
Architecture of the PMSPA-Net model.

During semantic segmentation, PMSPA-Net classified each point into one of three semantic categories: background, leaf, or stem. Dataset partitioning was performed at the whole-plant NPY file level before point-block sampling. Of the 65 complete plant samples, 15 were randomly selected once and retained as a fixed independent test set throughout all experiments. For each random seed, the remaining 50 samples were divided into training and validation sets at a 4:1 ratio, corresponding to 40 training samples and 10 validation samples. Point blocks were then generated independently within each subset, ensuring that blocks derived from the same plant could not occur in different subsets.

Each NPY file contained (X,Y,Z) and (R,G,B) features and one semantic-label column. For the common-protocol five-model comparison and the fixed-hyperparameter ablation configurations, each point block contained (Npoint = 4096) points. For the IOA-selected ablation configurations, the selected Npoint value was reported explicitly for each configuration.

#### 2.3.3 PMSPA-Net model hyperparameter optimization

The selection of hyperparameters during deep learning model training has a direct impact on model performance (Du et al., 2021). To address hyperparameter optimization for the proposed PMSPA-Net model in the 3D point cloud segmentation task, this study introduces a novel intelligent optimization algorithm—the Ivy Optimization Algorithm (IOA)—to improve both model performance and convergence efficiency. IOA is characterized by its strong global search capability and rapid convergence, inspired by the growth behavior of ivy plants to explore high-dimensional parameter spaces (Ghasemi et al., 2024). In this study, IOA is applied to optimize key hyperparameters of the PMSPA-Net model, including the learning rate, decay rate, Npoint, and batch size. Through IOA’s adaptive search mechanism, the inefficiencies of manual hyperparameter tuning commonly seen in traditional methods can be effectively avoided. The model integrated with the hyperparameter *o*ptimization algorithm is referred to as PMSPA-Net, and its architecture is illustrated in **Figure 3**.

IOA was used solely as a hyperparameter optimization strategy and was not incorporated into the network architecture. The same search space was applied to PointNet++ and PointNet++ + MSPA, including a learning rate ranging from 2×10^−4^ to 3×10^−3^, a weight decay ranging from 1×10^−6^ to 1×10^−3^, *N*_point_ ranging from 1024 to 4096, and a batch size ranging from 2 to 8. Each optimization run used a population size of 6, three iterations, five warm-up training epochs, and four training blocks per plant sample. A total of 63 objective-function evaluations were recorded. The optimization objective was defined as shown in **Equation 1**:

(1) $$J=\left(1-mIoU_{train}\right)+0.02{ℒ}_{train}$$

where ${ℒ}_{\text{train}}$ denotes the training loss. The fixed independent test set was not used during hyperparameter optimization.

For PointNet++, IOA selected a learning rate of 2.028×10^−3^, a weight decay of 2.990×10^−4^, *N*_point_ = 4096, and a batch size of 4. For PointNet++ + MSPA, the corresponding selected values were 1.982×10^−3^, 7.528×10^−4^, *N*_point_ = 4096, and a batch size of 8. The selected configurations were subsequently retrained from scratch for 300 epochs using random seeds 2026, 2027, and 2028. Model parameter counts and training times under the common hardware environment are su***m***marized in **Table 1**. Training times are reported as mean ± standard deviation across the three random seeds.

**Table 1:** Model complexity and training time of the five semantic segmentation models under the common experimental protocol.

Model	Parameters (M)	Training time (min)
PointNet	0.273	8.3 ± 0.3
PointNet++	0.967	36.4 ± 2.4
PMSPA-Net	1.740	35.6 ± 0.3
DGCNN	1.773	51.2 ± 0.1
RandLA-Net	0.648	37.7 ± 0.3

#### 2.3.4 Model performance evaluation

All experiments followed the whole-plant dataset-partitioning protocol described in Section 2.3.1. Point blocks were generated independently within the training, validation, and test subsets to prevent blocks derived from the same plant from appearing in different subsets.

A two-factor, two-level (2×2) ablation study was conducted to evaluate the individual and combined effects of the MSPA module and IOA-based hyperparameter optimization. The four configurations were: (1) PointNet++ with fixed hyperparameters, (2) PointNet++ + MSPA with the same fixed hyperparameters, (3) PointNet++ with IOA-selected hyperparameters, and (4) PointNet++ + MSPA with IOA-selected hyperparameters. All final configurations were trained for 300 epochs using random seeds 2026, 2027, and 2028 and were evaluated on the same fixed independent test set.

The baseline model comparison followed the dataset-partitioning protocol described in Section 2.3.1. PointNet, PointNet++, PMSPA-Net, DGCNN, and RandLA-Net were evaluated under identical experimental settings. All models were trained for 300 epochs, and the resulting final checkpoints were evaluated on the fixed independent test set. Model performance was assessed using overall accuracy (OA), mean intersection over union (mIoU), macro-averaged F1-score (macro-F1), balanced accuracy, class-specific IoU, precision, recall, F1-score, and confusion matrices.

Let *N* denote the total number of evaluated points and *C* the number of semantic classes. For the *i*-th point, *y_i_* denotes the ground-truth class label and ${\hat{y}}_{i}$ denotes the predicted class label. Overall accuracy was calculated using **Equation 2**:

(2) $$OA=\frac{1}{N}\sum_{i=1}^{N}I\left({\hat{y}}_{i}=y_{i}\right)$$

where the indicator function is defined in **Equation 3**:

(3) $$I\left({\hat{y}}_{i}=y_{i}\right)=\{\begin{array}{cc} 1, & {\hat{y}}_{i}=y_{i},\\ 0, & {\hat{y}}_{i}\neq y_{i}. \end{array}$$

For class c, the class-specific intersection over union was calculated using **Equation 4**:

(4) $$IoU_{c}=\frac{TP_{c}}{TP_{c}+FP_{c}+FN_{c}}$$

where *TP*_c_, *FP_c_*, and *FN_c_* denote the numbers of true-positive, false-positive, and false-negative predictions for class *c*, respectively. The mIoU was obtained by averaging the IoU values across all semantic classes, as shown in **Equation 5**:

(5) $$mIoU=\frac{1}{C}\overset{C}{\underset{c=1}{\sum }}\frac{TP_{c}}{TP_{c}+FP_{c}+FN_{c}}$$

Class-specific precision, recall, and F1-score were calculated using **Equations 6**–**8**:

(6) $$Precision_{c}=\frac{TP_{c}}{TP_{c}+FP_{c}}$$

(7) $$Recall_{c}=\frac{TP_{c}}{TP_{c}+FN_{c}}$$

(8) $$F1_{c}=\frac{2\times Precision_{c}\times Recall_{c}}{Precision_{c}+Recall_{c}}$$

Macro-F1 and balanced accuracy were defined as the arithmetic means of the class-specific F1-scores and recalls, respectively, as shown in **Equations 9**, **10**:

(9) $$Macro-F1=\frac{1}{C}\sum_{c=1}^{C}F1_{c}$$

(10) $$Balanced\;Accuracy=\frac{1}{C}\sum_{c=1}^{C}Recall_{c}$$

A class-weighted negative log-likelihood loss, as defined in **Equation 11**, was used for model training:

(11) $${ℒ}_{NLL}=-\frac{1}{N}\sum_{i=1}^{N}w_{y_{i}}\log\left(p_{i,y_{i}}\right)$$

where $p_{i,y_{i}}$ denotes the predicted probability that point ***i*** belongs to its ground-truth class *y_i_*, and $w_{y_{i}}$ is the weight assigned to that class. Because background points accounted for a large proportion of the maize seedling point clouds, reliance on *OA* alone could overestimate segmentation performance. Therefore, *mIoU*, *Macro* – *F*1, *BalancedAccuracy*, and class-specific metrics were reported alongside *OA* to reduce evaluation bias associated with the dominant background class.

#### 2.3.5 Maize point cloud organ segmentation

In plant phenotyping research, applying clustering algorithms for organ-level segmentation of 3D point clouds has become a common approach in the field (Elnashef et al., 2019). Clustering algorithms group point clouds into distinct clusters by computing object features such as normal vectors and color information. However, when dealing with plant point clouds, clustering methods still face two major limitations. On one hand, maize leaves typically exhibit a uniformly green appearance, which significantly limits the effectiveness of using color features as clustering criteria (Pascucci et al., 2018). On the other hand, overlapping between maize leaf point clouds is often unavoidable, substantially increasing the complexity of segmentation and posing additional challenges to the performance of clustering algorithms (Miao et al., 2023).

Conventional clustering methods cannot reliably separate adhered leaves at the organ level. To address this limitation, we developed a geometric region decomposition algorithm for individual-leaf segmentation based on the outputs of PMSPA-Net. Semantic segmentation first classified the point cloud into background, leaf, and stem classes, after which only the leaf points were retained for organ-level decomposition. As shown in **Figure 4**, the proposed algorithm uses AlphaShape-based contour extraction to identify leaf-boundary points, applies region-growing connectivity analysis to separate candidate boundary regions, and performs geometric envelope clipping to divide adhered leaves into complete individual organs. The principal parameters used for AlphaShape extraction, region growing, envelope clipping, and the comparative DBSCAN method are summarized in **Table 2**. By exploiting boundary geometry rather than relying solely on point density, the proposed workflow improves the separation of adjacent leaves that remain partially connected.

**Figure 4:**
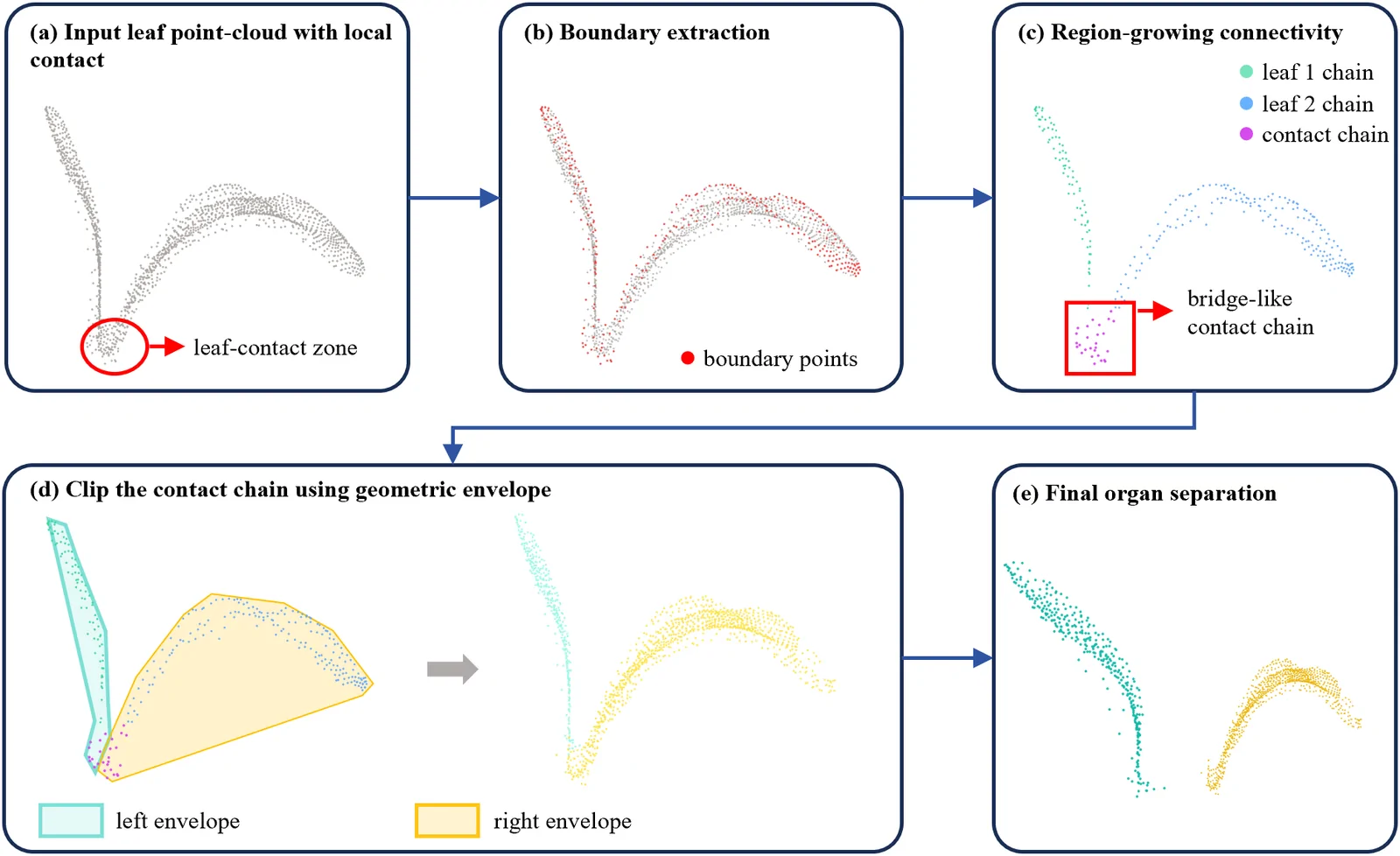
Geometric region decomposition workflow for separating adhered maize leaves. **(a)** Input leaf point-cloud with an adhered region. **(b)** AlphaShape-based boundary extraction. **(c)** Region-growing connectivity analysis of boundary components. **(d)** Geometric envelope clipping of the adhered region. **(e)** Final separation of individual leaf organs.

**Table 2:** Parameter settings for the proposed geometric region decomposition algorithm and the comparative DBSCAN method.

Method	Parameter values
AlphaShape	alpha = 25; rotations around x/y/z axes; 10 angles; retained when detected in at least 6 projections
Region growing	Search radius = 0.05; minimum connected-component size = 20 points
Envelope clipping	3D ConvexHull + Delaunay; points satisfying find_simplex >= 0 are retained
DBSCAN	eps = 0.04; minPts = 12; minimum cluster size = 32 points

### 2.4 High-precision phenotypic trait extraction

Organ-level segmentation of field-grown maize seedlings was performed by combining PMSPA-Net with the geometric region decomposition algorithm. The separated leaf point clouds were used to extract five phenotypic traits: leaf length, leaf width, leaf edge contour, leaf margin color, and leaf curl degree.

Traditional methods for measuring leaf morphology—such as 2D projection-based approaches—offer advantages in simplicity and efficiency but present significant limitations in capturing the three-dimensional structural characteristics of leaves. These limitations become particularly pronounced when leaves exhibit bending or curling, often resulting in inaccuracies in area and shape measurements (Paulus, 2019). To address these challenges, Ryuhei Ando et al. developed a leaf flattening algorithm based on 3D plant point clouds (Ando et al., 2021). This algorithm first defines the primary axis (aligned with the main vein, representing the leaf’s longitudinal direction) and the secondary axis (aligned with the secondary veins, representing the leaf’s width direction). It then extracts the leaf skeleton, including both main and secondary veins. The algorithm progressively corrects for bending along the primary axis and curling along the secondary axis, transforming the complex 3D morphology into a flattened 2D representation. By decoupling the deformation and flattening processes, the method greatly simplifies leaf surface reconstruction and enhances robustness in the presence of noise and missing data. In this study, we applied this algorithm to flatten each segmented maize leaf onto a 2D plane. During the flattening process, the curvature angles along both the primary and secondary axes were recorded as quantitative indicators of leaf curl degree. After flattening, the leaf width was defined as the distance between the two farthest points along the x-axis, while leaf length was calculated as the distance between the two farthest points along the y-axis. To obtain leaf size parameters with real biological relevance, the Euclidean distances in the point cloud coordinate space were converted to physical units. To this end, a checkerboard with known physical dimensions (1.5 cm × 1.5 cm) was introduced as a reference during 3D point cloud reconstruction. Considering potential distortions in multi-view imagery, we extracted Euclidean distances along the checkerboard edges that were aligned with the leaf length and width directions, using these as spatial calibration coefficients for leaf length and width. This calibration process ensures an accurate mapping from digital space to the physical world. The specific formulas for calculating leaf length and leaf width are presented in Equations 12–14. Here, ϵ represents the spatial calibration coefficient, *Leaf_width_* denotes leaf width, *Leaf_length_* denotes leaf length, and *x_i_* and *y_i_* refer to the x and y coordinates of the flattened leaf point cloud, respectively.

(12) $$\epsilon =\frac{Reference\;physical\;value}{Reference\;euclidean\;distance}$$

(13) $$Leaf_{width}=\epsilon_{2}\times \left(max\left(x_{i}\right)-\min\left(x_{i}\right)\right)$$

(14) $$Leaf_{length}=\epsilon_{1}\times \left(max\left(y_{i}\right)-min\left(y_{i}\right)\right)$$

The AlphaShape method was used to extract boundary contours from each leaf point cloud. Because leaf-edge curvature reflects local bending along the margin and captures edge smoothness or waviness, curvature was calculated for the extracted edge contours using Equation 15. In this equation, *K* denotes curvature; *x′*, *y′*, and *z′* represent the first-order derivatives of the edge contour in the *x*, *y*, and z directions; and *x″*, *y″*, and *z″* represent the corresponding second-order derivatives.

(15) $$K=\frac{\sqrt{{\left(y^{\prime }z^{\prime \prime }-z^{\prime }y^{\prime \prime }\right)}^{2}+{\left(z^{\prime }x^{\prime \prime }-x^{\prime }z^{\prime \prime }\right)}^{2}+{\left(x^{\prime }y^{\prime \prime }-y^{\prime }x^{\prime \prime }\right)}^{2}}}{{\left(x^{\prime 2}+y^{\prime 2}+z^{\prime 2}\right)}^{3/2}}$$

Finally, leaf-margin color was obtained directly from the RGB channels of the original XYZRGB point cloud. Mean margin RGB is reported as a descriptive color value rather than a validated measure of color heterogeneity. Leaf-edge curvature was calculated from the extracted boundary but had no independent ground-truth measurement; it is therefore interpreted only as a computed geometric descriptor.

## 3 Results

### 3.1 PMSPA-Net ablation experiment

A 2×2 ablation experiment was conducted to evaluate the effects of the MSPA module and IOA-based hyperparameter optimization. The quantitative results are summarized in **Table 3**, and **Figure 5** compares the overall metrics, class-specific IoU values, and performance variation across random seeds.

**Table 3:** Ablation results for the MSPA module and IOA-based hyperparameter optimization. Values are reported as mean ± standard deviation across three random seeds.

Model	OA	mIoU	Macro-F1	Balanced accuracy	IoU leaf	IoU stem
PointNet++ baseline	0.9930± 0.0061	0.7442± 0.0674	0.8393± 0.0734	0.8722± 0.0632	0.7395± 0.1959	0.5001± 0.0002
PointNet++ + MSPA	0.9962± 0.0015	0.7856± 0.0252	0.8669± 0.0225	0.9141± 0.0251	0.8203± 0.0607	0.5419± 0.04512
PointNet++ + IOA	0.9974± 0.0008	0.7958± 0.0182	0.8766± 0.0161	0.9284± 0.0188	0.8011± 0.0544	0.5926± 0.0351
PMSPA-Net full	0.9981± 0.0020	0.8286± 0.0149	0.8981± 0.0238	0.9561± 0.0223	0.8601± 0.0501	0.6267± 0.0131

**Figure 5:**
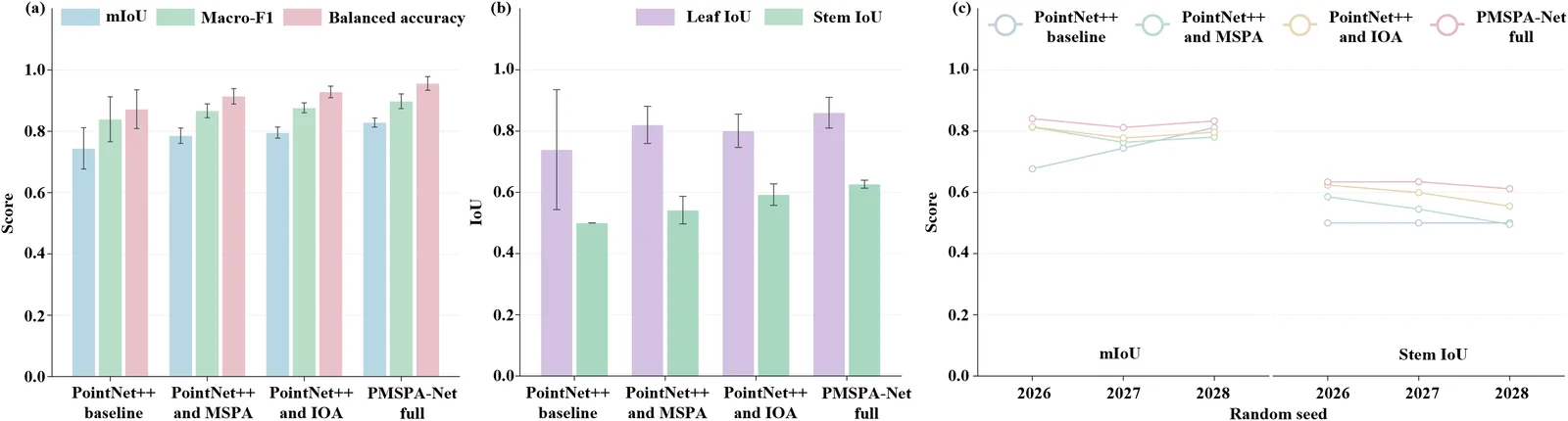
Ablation analysis of the MSPA module and IOA-based hyperparameter optimization. **(A)** Comparison of mIoU, macro-F1, and balanced accuracy; **(B)** comparison of leaf and stem IoU; and **(C)** variation in mIoU and stem IoU across three random seeds. Bars represent mean values, and error bars indicate standard deviations.

Compared with the PointNet++ baseline, adding MSPA increased mIoU from 0.7442 ± 0.0674 to 0.7856 ± 0.0252, whereas IOA increased it to 0.7958 ± 0.0182. The complete PMSPA-Net achieved the best performance, with an mIoU of 0.8286 ± 0.0149, a macro-F1 of 0.8981 ± 0.0238, and a balanced accuracy of 0.9561 ± 0.0223. It also obtained the highest leaf IoU (0.8601 ± 0.0501) and stem IoU (0.6267 ± 0.0131). As shown in **Figure 5**, MSPA mainly improved leaf segmentation, whereas IOA produced a greater improvement in stem segmentation. Their combination yielded the highest and most stable performance across the three random seeds. Because OA exceeded 0.99 for all configurations, the ablation analysis focused primarily on mIoU, macro-F1, balanced accuracy, and class-specific IoU.

### 3.2 Semantic segmentation comparison

All experiments in this study were conducted on a computer equipped with an Intel^®^ Core™ i7-13700KF CPU, an RTX 4070 Ti SUPER (16GB) GPU, and 32 GB RAM. The experimental environment was based on the Windows 11 operating system and implemented using the PyTorch framework.

Under the common evaluation protocol, PMSPA-Net achieved the highest mIoU of 0.8286 ± 0.0149, followed by RandLA-Net (0.7787 ± 0.0363), PointNet++ (0.7442 ± 0.0674), DGCNN (0.7296 ± 0.0269), and PointNet (0.6979 ± 0.0860). PMSPA-Net also achieved the highest macro-F1 (0.8981 ± 0.0238), balanced accuracy (0.9561 ± 0.0223), leaf IoU (0.8601 ± 0.0501), and stem IoU (0.6267 ± 0.0131). Although OA exceeded 0.99 for all five models, the class-specific results revealed substantial differences in leaf and stem segmentation performance, confirming that OA alone was insufficient to distinguish model performance. Detailed results are presented in **Table 4**.

**Table 4:** Semantic segmentation performance of five baseline models on the fixed independent test set under a common evaluation protocol.

Model	OA	mIoU	Macro-F1	Balanced accuracy	Background IoU	Leaf IoU	Stem IoU
PointNet	0.9985± 0.0010	0.6979± 0.0860	0.7791± 0.0774	0.7740± 0.0505	0.9987± 0.0010	0.7886± 0.1478	0.3064± 0.1113
PointNet++	0.9930± 0.0061	0.7442± 0.0674	0.8393± 0.0734	0.8722± 0.0632	0.9930± 0.0062	0.7395± 0.1959	0.5001± 0.0002
PMSPA-Net	0.9981± 0.0020	0.8286± 0.0149	0.8981± 0.0238	0.9561± 0.0223	0.9992± 0.0019	0.8601± 0.0501	0.6267± 0.0131
DGCNN	0.9983± 0.0005	0.7296± 0.0269	0.8207± 0.0227	0.8543± 0.0194	0.9985± 0.0005	0.7634± 0.0316	0.4270± 0.0508
RandLA-Net	0.9987± 0.0003	0.7787± 0.0363	0.8614± 0.0308	0.8803± 0.0318	0.9990± 0.0003	0.7964± 0.0296	0.5409± 0.1015

All models were trained for 300 epochs using Npoint = 4096 and random seeds 2026, 2027, and 2028. Values are reported as mean ± standard deviation.

The fixed independent test set contained 3,835,046 background points, 29,986 leaf points, and 2,468 stem points. **Table 5** therefore reports sample-level class recall rather than class accuracy. Across the 15 test samples, background recall ranged from 0.9986 to 0.9999, leaf recall from 0.9262 to 0.9895, and stem recall from 0.8554 to 0.9820. The point-count-weighted recalls for background, leaf, and stem were 0.9996, 0.9550, and 0.9137, respectively, corresponding to a balanced accuracy of approximately 0.9561. Stem recall showed the greatest sample-to-sample variation, confirming that the comparatively sparse stem class remained the most challenging category.

**Table 5:** Sample-level class recalls and point distributions for PMSPA-Net on the fixed independent test set.

Index	Background recall	Leaf recall	Stem recall	Number of backgrounds	Number of leaf	Number of stem
1	0.9986	0.9269	0.8596	263588	1593	180
2	0.9998	0.9889	0.9421	263588	1593	180
3	0.9998	0.9269	0.8849	264612	1437	110
4	0.9999	0.9269	0.9572	264374	2102	198
5	0.9999	0.9265	0.8664	266387	2112	106
6	0.9996	0.9833	0.8971	247796	1829	186
7	0.9991	0.9844	0.9820	246889	2595	180
8	0.9994	0.9551	0.9489	237536	1660	182
9	0.9997	0.9576	0.9345	242731	2294	142
10	0.9994	0.9546	0.8864	260730	1794	114
11	0.9999	0.9263	0.9114	249558	2248	103
12	0.9998	0.9579	0.8554	259515	2353	161
13	0.9999	0.9796	0.9745	257894	2304	177
14	0.9995	0.9895	0.9349	253659	2137	275
15	0.9998	0.9262	0.8839	256189	1935	174

**Figure 6**, summarizes the quantitative results and training behavior. PMSPA-Net achieved the strongest class-specific and overall test metrics. The weighted cross-entropy loss decreased progressively for all models during training. Although DGCNN and RandLA-Net reached lower final training losses, PMSPA-Net produced the highest test mIoU, macro-F1, and balanced accuracy, indicating that terminal training loss alone did not determine generalization performance.

**Figure 6:**
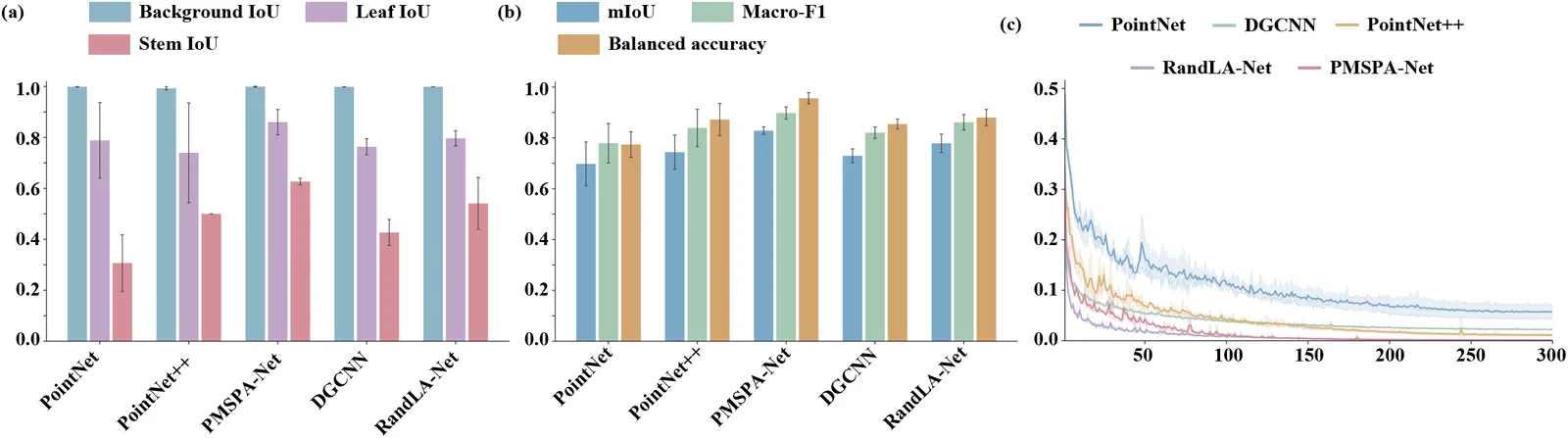
Quantitative performance and training-loss comparison of the five semantic segmentation models. **(A)** Class-specific IoU values; **(B)** overall evaluation metrics; and **(C)** class-weighted cross-entropy loss over 300 training epochs. Bars represent mean values, error bars indicate standard deviations across three random seeds, and shaded regions in panel **(C)** indicate inter-run variation.

The qualitative comparisons in **Figure 7** further support the quantitative results. PointNet and PointNet++ showed more omission, redundancy, and boundary confusion near slender stems and leaf–stem junctions. PMSPA-Net preserved the stem structures more consistently and produced clearer boundaries among background, leaf, and stem points, particularly in the enlarged regions.

**Figure 7:**
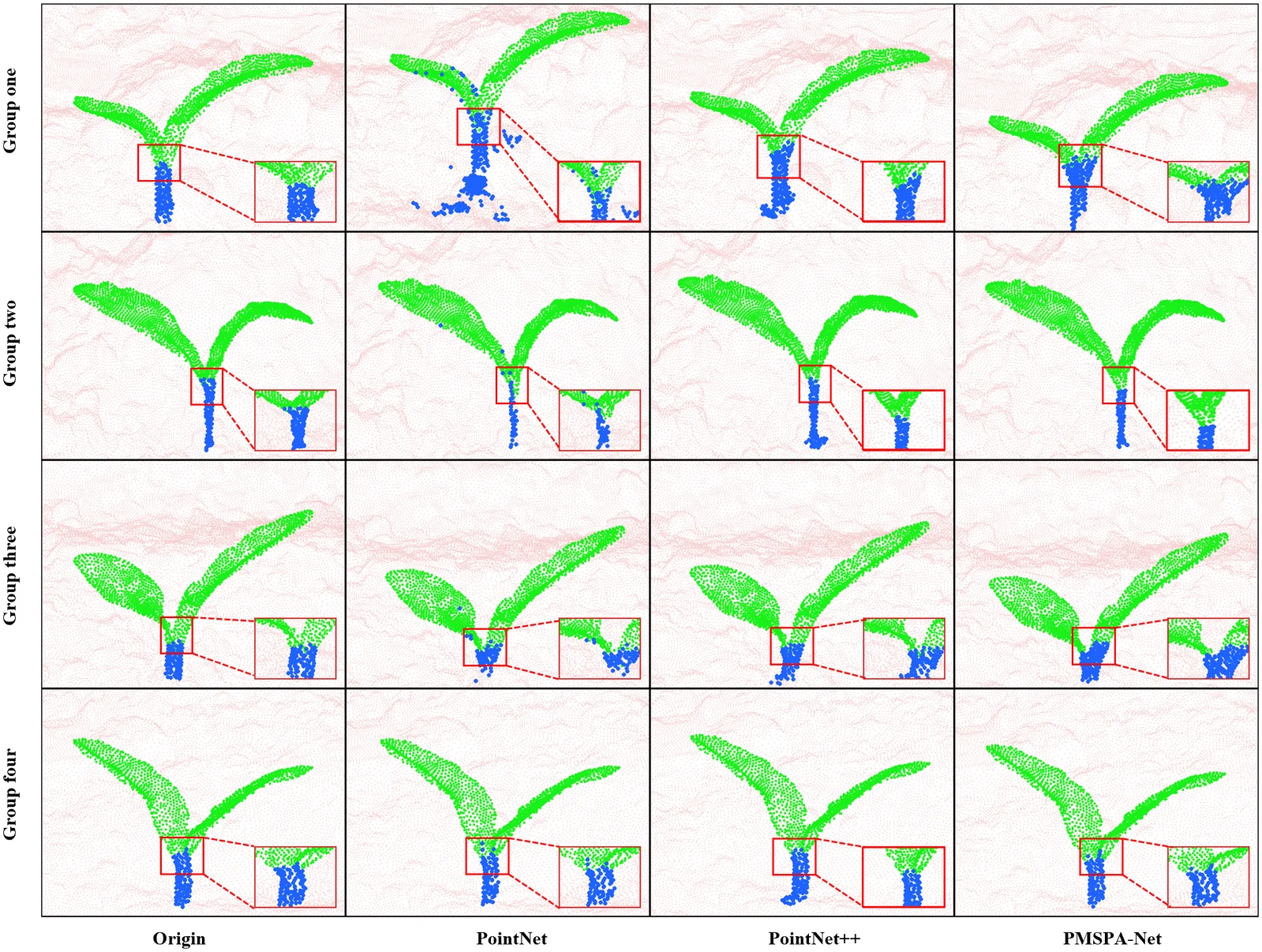
Qualitative comparison of semantic segmentation results for representative maize seedling point clouds. Rows represent different test samples, and columns show the ground truth, PointNet, PointNet++, and PMSPA-Net results. Red boxes and enlarged insets highlight stem and leaf-base regions where omission, redundancy, and boundary confusion are most evident.

### 3.3 Results of organ-level segmentation based on geometric region decomposition

**Figure 8** presents qualitative comparisons of adhered-leaf separation for three representative point-cloud samples. The reference labels, GRD results, and DBSCAN results are shown from left to right. In all three cases, GRD correctly separated the two adhered leaves and closely reproduced the reference instance boundaries, including the curved leaf margins and contact regions. DBSCAN also identified the two dominant leaf clusters but showed local point loss and occasional fragmentation near leaf junctions and sparse boundary regions, with some points classified as noise. These observations are consistent with the quantitative results (**Figure 9**), in which GRD achieved higher ARI, homogeneity, completeness, and V-measure and produced no over- or under-segmentation. Overall, GRD provided more complete and stable leaf-instance separation under different adhesion geometries.

**Figure 8:**
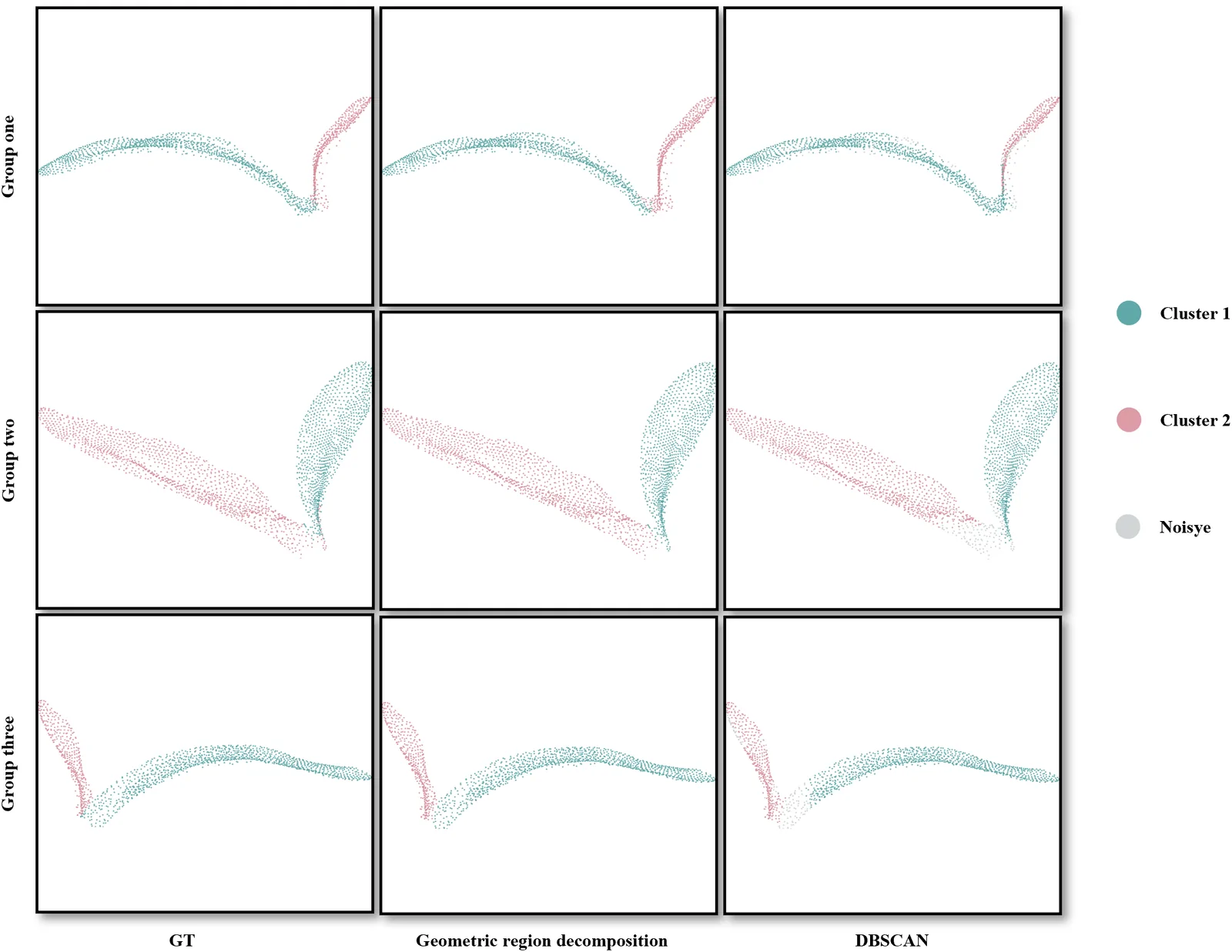
Qualitative comparison of adhered-leaf separation results. Rows represent three adhered-leaf cases, and columns show the reference instance labels, geometric region decomposition results, and DBSCAN results. Different colors indicate individual leaf clusters, and gray points indicate noise identified by DBSCAN.

**Figure 9:**
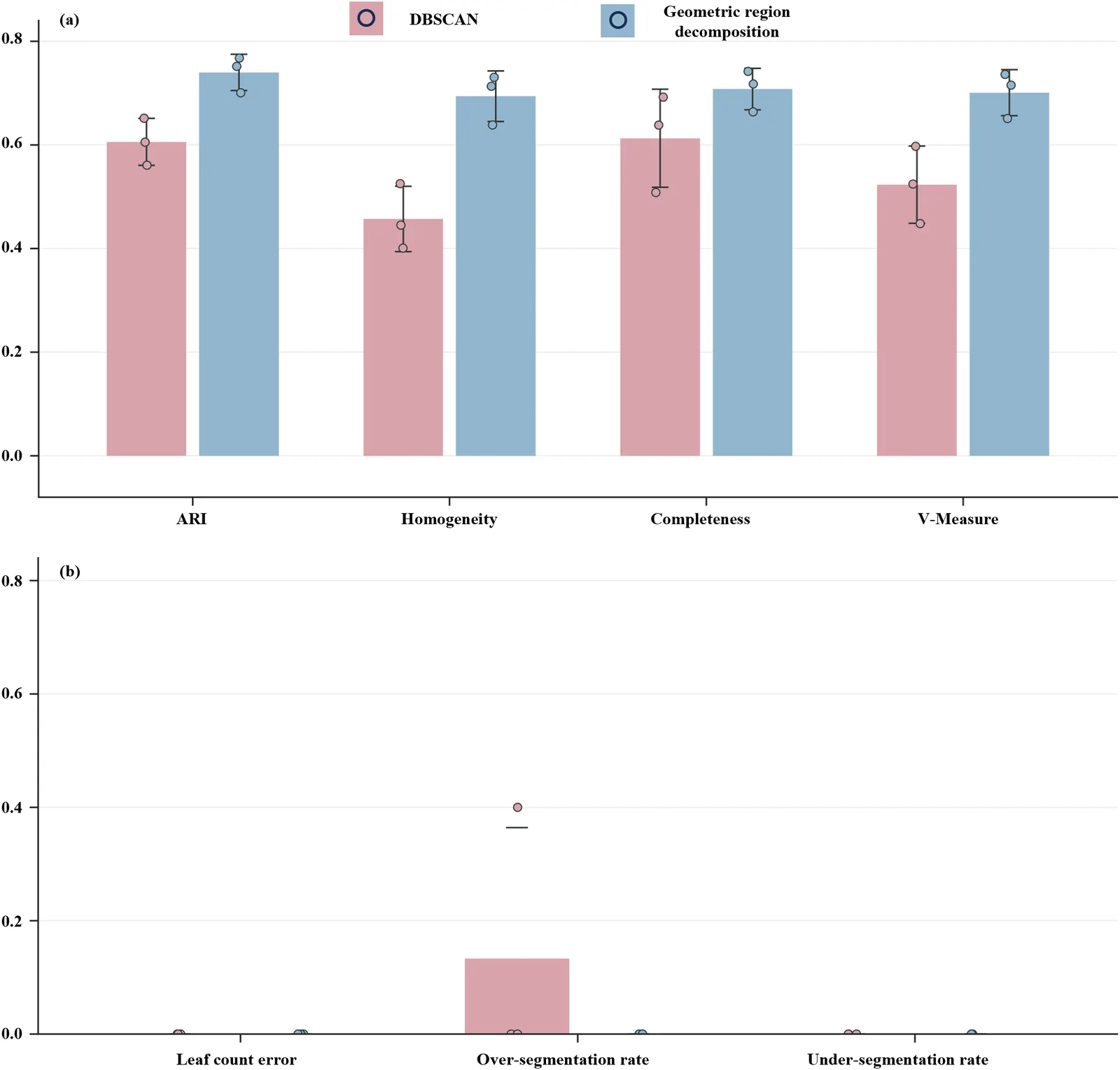
Quantitative comparison of DBSCAN and the proposed geometric region decomposition method for adhered-leaf separation. **(A)** Adjusted Rand index (ARI), homogeneity, completeness, and V-measure; and **(B)** leaf-count error, over-segmentation rate, and under-segmentation rate. Bars represent mean values across three annotated cases, error bars indicate standard deviations, and circles represent individual cases.

**Figure 9** quantitatively compares the proposed geometric region decomposition (GRD) method with DBSCAN. As shown in **Figure 9A**, GRD consistently outperformed DBSCAN in all clustering-agreement metrics, achieving higher ARI (0.925 ± 0.044), homogeneity (0.885 ± 0.050), completeness (0.868 ± 0.061), and V-measure (0.876 ± 0.056). In comparison, the corresponding values for DBSCAN were 0.757 ± 0.057, 0.766 ± 0.118, 0.571 ± 0.079, and 0.654 ± 0.093, respectively. As shown in **Figure 9B**, neither method produced leaf-count errors or under-segmentation. However, DBSCAN exhibited an over-segmentation rate of 0.167 ± 0.289, whereas no over-segmentation was observed for GRD. These results indicate that GRD achieved more accurate and stable separation of adhered leaves than DBSCAN.

### 3.4 Results of phenotypic parameter extraction

**Figure 10** shows good agreement between the point-cloud-derived measurements and the corresponding manual measurements. Leaf length achieved an R^2^ of 0.88 and an RMSE of 0.56 cm, with most observations distributed close to the 1:1 reference line across the measured range (**Figure 10A**). Leaf width also showed a strong relationship with manual measurements, with an R^2^ of 0.83 and an RMSE of 0.10 cm (**Figure 10B**). Although some dispersion was observed, particularly within the intermediate measurement ranges, no pronounced systematic deviation from the 1:1 line was evident. These results indicate that the reconstructed point clouds can provide reliable estimates of maize leaf length and width, with leaf length showing a slightly stronger correlation and leaf width exhibiting a lower absolute prediction error.

**Figure 10:**
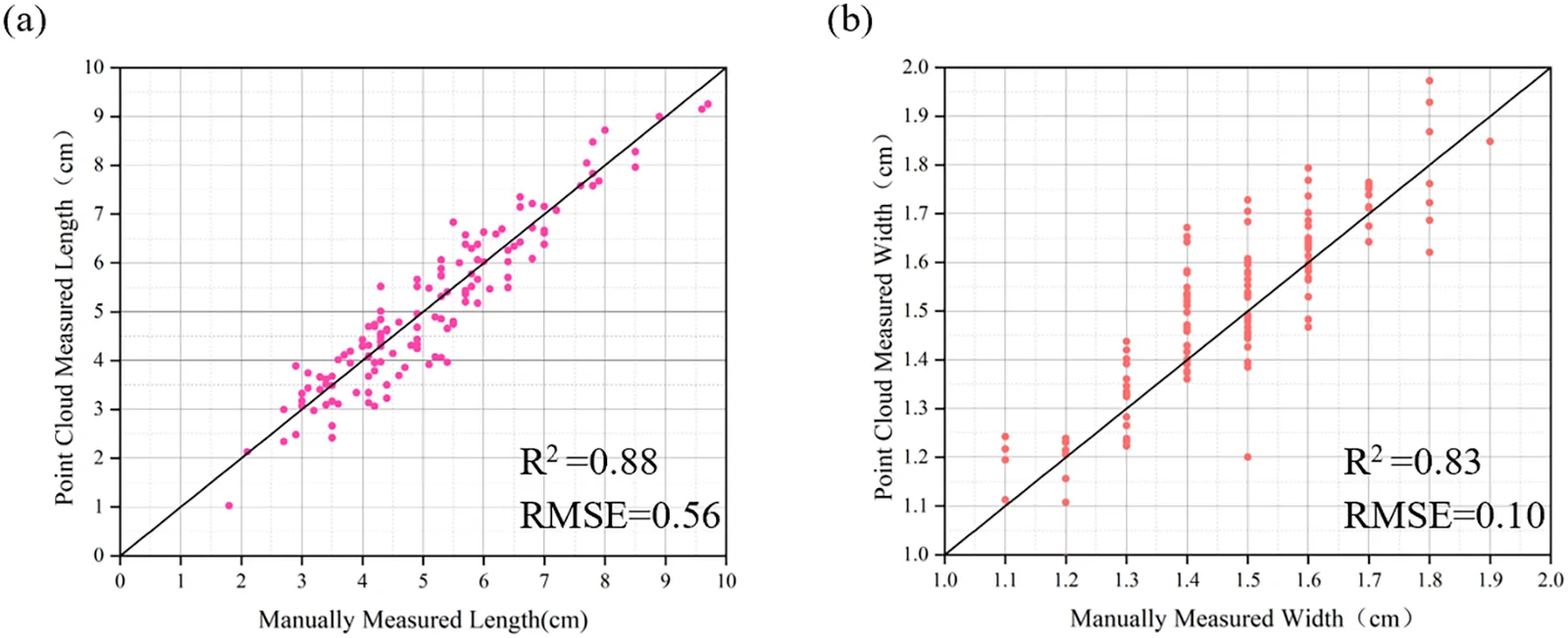
Regression analysis between manually measured and point-cloud-derived maize seedling leaf dimensions. **(A)** Leaf length. **(B)** Leaf width.

**Figure 11** summarizes leaf-margin phenotypes extracted from the segmented maize seedling point clouds. The edge-curvature atlas (**Figure 11A**) shows variation in local curvature across 12 representative leaf clusters, and the detailed examples in **Figure 11B** compare two leaves from case 1. The first highlighted leaf contained 109 edge points, an edge length of 11.9, and a curvature standard deviation of 16.50, whereas the second contained 198 edge points, an edge length of 18.5, and a curvature standard deviation of 22.71. Curvature-density curves (**Figure 11C**) show that the two leaves differed in the height and width of their low-curvature peaks. The group-level distributions (**Figure 11D**) and trait-space plot (**Figure 11E**) place these examples within the broader population, indicating that edge length and curvature variability jointly capture differences in leaf-margin morphology. However, leaf curl degree, quantified as curvature angles along the primary and secondary axes during the flattening process, was not validated against independent manual measurements and is therefore reported here as a computed geometric descriptor rather than a validated phenotypic trait.

**Figure 11:**
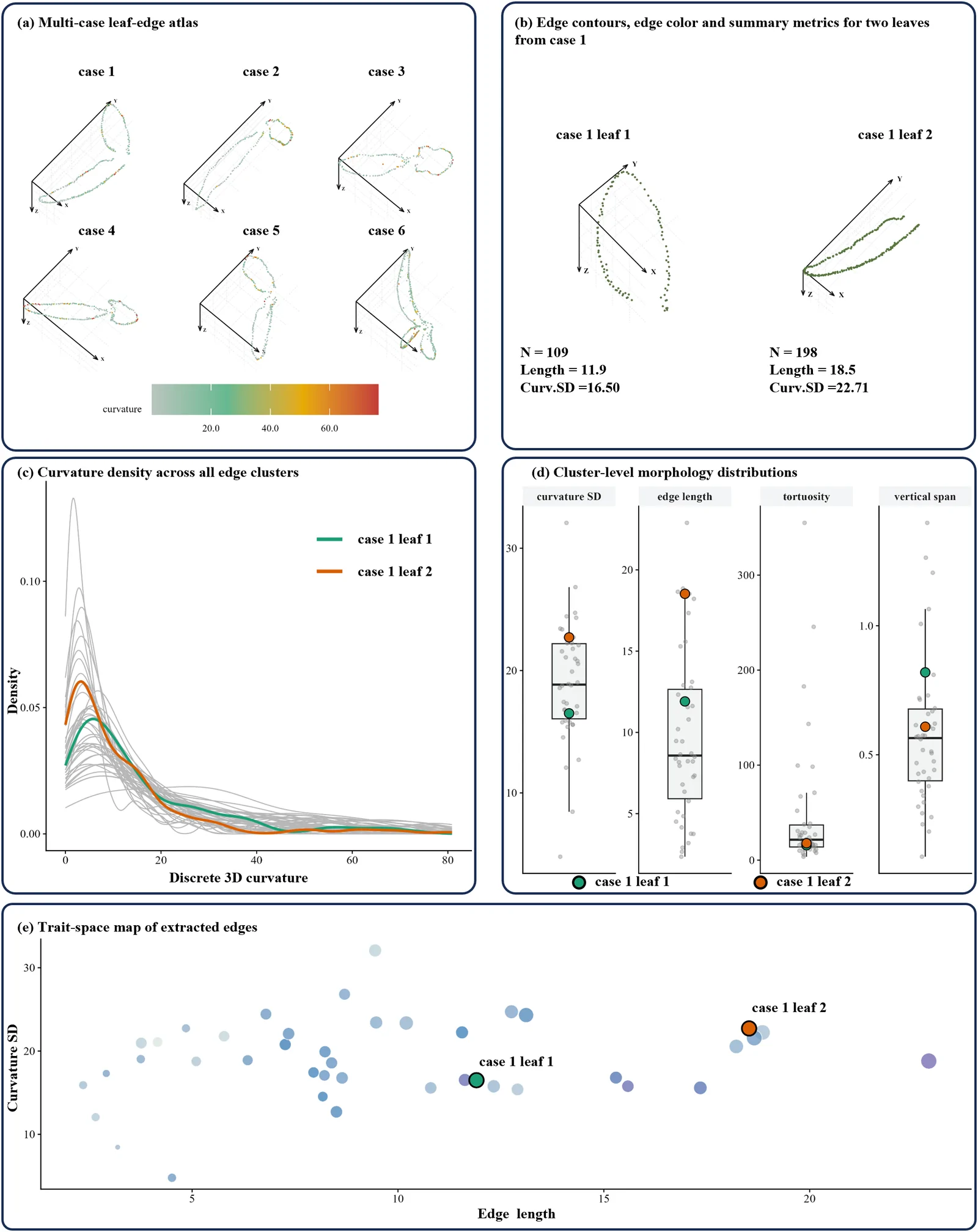
Leaf-margin phenotypes extracted from maize seedling point clouds. **(A)** Edge-curvature atlas for 12 representative leaf clusters, with point color indicating discrete 3D curvature. **(B)** Edge contours, edge color and summary metrics for two leaves from case 1. **(C)** Curvature-density curves across edge clusters, with the two case-1 leaves highlighted. **(D)** Distributions of curvature standard deviation, edge length, tortuosity, and vertical span; colored points correspond to the highlighted case-1 leaves. **(E)** Two-dimensional trait space based on edge length and curvature standard deviation.

## 4 Discussion

### 4.1 Construction of high-quality field plant point clouds

High-quality point clouds are important for semantic segmentation and phenotypic trait extraction, but field-based multi-view reconstruction remains challenging because of complex backgrounds, variable illumination, and plant movement (Li et al., 2021; Wu et al., 2024). In this study, multi-view RGB images and OpenMVS were used to reconstruct colored point clouds of maize seedlings under the recorded acquisition conditions. The reconstructed point clouds preserved the main structural characteristics of leaves and stems and provided the basis for subsequent semantic segmentation, adhered-leaf separation, and extraction of geometric descriptors. However, slight leaf movement during image acquisition caused cross-view inconsistencies and local reconstruction artefacts, particularly near leaf tips, margins, and slender stems.

As shown in **Table 5**, stem recall exhibited the largest sample-to-sample variation among the three classes. This variability was likely related to both reconstruction quality and plant architecture. Compared with broad leaf surfaces, slender stems provide fewer distinctive visual features for multi-view matching and are more susceptible to occlusion by surrounding leaves, which can reduce the density and completeness of reconstructed stem points. Misclassification may also occur near leaf–stem junctions, where curved leaf surfaces exhibit local geometric characteristics similar to those of stems. Differences in leaf angle, leaf–stem overlap, and reconstruction completeness among individual plants further contribute to variation in stem visibility and point density. These factors help explain the greater fluctuation in stem recall observed across the test samples.

The present study was conducted at a single site, during one acquisition period, and at the seedling stage under clear-weather conditions. Moreover, independent reconstruction-accuracy measurements, reconstruction failure rates, and complete processing-time records were unavailable. The results should therefore be interpreted as demonstrating the feasibility of the workflow under the evaluated conditions rather than establishing broad robustness, high-throughput performance, or field scalability. Future studies should incorporate systematic records of reconstruction success and processing time, and datasets collected across different sites, developmental stages, dates, and illumination conditions. The integration of complementary sensing technologies, such as LiDAR or hyperspectral imaging, may further improve structural completeness and support more comprehensive field phenotyping.

### 4.2 Point cloud segmentation from semantic classes to individual organs

To address the vulnerability of standard models in real-world settings (Kamann and Rother, 2021), we introduced PMSPA-Net, which augments PointNet++ with a Multi-Scale Spatial Pyramid Attention (MSPA) module and the Ivy Optimization Algorithm (IOA). This design improves robustness to noise, occlusion, and class imbalance while preserving morphological structure. Quantitatively, the model achieved higher mean Intersection over Union (mIoU) than PointNet and PointNet++ at comparable overall accuracy, and category-wise analysis (**Table 4**) confirmed improved performance on minority classes.

In addition, the geometric region decomposition (GRD) algorithm separated fused leaves and enabled reliable organ-level segmentation. As a training-free geometric approach, GRD does not require additional instance-level annotations or model retraining, allowing it to be readily transferred to new datasets. Its reliance on Euclidean distance, normal orientation, and density-based clustering also keeps computational requirements relatively low compared with deep learning-based instance segmentation frameworks. Moreover, the decomposition process remains highly interpretable, as stem identification, spatial region partitioning, and subsequent leaf clustering can be directly inspected and adjusted according to species-specific morphological characteristics. These properties make GRD a practical complement to semantic segmentation, particularly when annotated data are limited, rapid deployment is required, or computational resources are constrained. The resulting semantic-to-organ workflow supports leaf-level trait extraction and can further inform agronomic decisions related to planting density, canopy light-use efficiency, early growth-vigor screening, and structure-related risk assessment. Remaining challenges include dataset imbalance, with leaves and background greatly outnumbering stems, as well as imperfect boundary delineation under strong adhesion or severe leaf curling. Future work should integrate instance-aware learning and topology-preserving decomposition to maintain organ continuity in more complex adhesion scenarios and improve the scalability of the framework for field deployment.

### 4.3 Phenotypic trait extraction

Following organ-level separation, each leaf was flattened along its primary and secondary axes into two dimensions to extract classical traits (length and width) and edge-centric descriptors (edge contour, leaf margin color, and curl degree), while leaf-edge curvature was summarized using density curves (**Figure 11**). This step was designed to address the morphological irregularity of field-grown maize seedlings, where leaf curling, margin deformation, partial damage, and non-planar blade structures can reduce the reliability of conventional size-based phenotyping. Curvature standard deviation, edge length, tortuosity, and vertical span provide complementary descriptors of leaf-margin morphology. Smooth leaves tend to occupy lower-curvature regions, whereas curled or damaged margins shift toward broader or multimodal curvature-density profiles (Rahadiyan et al., 2023). Agronomically, these descriptors complement length and width by supporting early quantitative assessment of stress-related morphology and targeted field interventions. However, leaf curl degree, derived from curvature angles computed during the flattening process, was not validated against independent manual assessments or reference samples with known levels of curling severity. It should therefore be interpreted as a computed geometric descriptor rather than a fully validated phenotypic trait. Future studies should establish its biological relevance through expert-scored curl ratings, comparisons among genotypes with contrasting curling responses, or repeated measurements under controlled dehydration treatments.

## 5 Conclusion

This study developed an integrated workflow that combines PMSPA-Net with geometric region decomposition for semantic and organ-level segmentation of field-grown maize seedling point clouds. Using a fixed whole-plant independent test set and three predefined random seeds, PMSPA-Net achieved the highest performance among the five evaluated models, with an mIoU of 0.8286 ± 0.0149, a macro-F1 of 0.8981 ± 0.0238, a balanced accuracy of 0.9561 ± 0.0223, a leaf IoU of 0.8601 ± 0.0501, and a stem IoU of 0.6267 ± 0.0131. The ablation analysis showed that MSPA primarily improved leaf segmentation, whereas IOA-based hyperparameter optimization contributed more strongly to stem segmentation; their combination produced the best overall and most stable performance.

Geometric region decomposition separated adhered leaves more accurately and completely than DBSCAN across three annotated cases and provided organ-level inputs for downstream phenotyping. Point-cloud-derived leaf length and width showed good agreement with manual measurements, while leaf-edge and curl descriptors provided additional geometric characterization. The study demonstrates feasibility under the evaluated single-site, seedling-stage conditions. Further work should assess reconstruction accuracy, processing time, and generalization across sites, growth stages, illumination conditions, and larger instance-level datasets.

## Data Availability

The raw data supporting the conclusions of this article will be made available by the authors, without undue reservation.
